# Carotid Arterial Compliance during Different Intensities of Submaximal Endurance Exercise

**DOI:** 10.3390/jcm13113316

**Published:** 2024-06-04

**Authors:** Alvaro N. Gurovich, Samuel Montalvo, Progga F. Hassan, Manuel Gomez

**Affiliations:** 1 Clinical Applied Physiology Laboratory, College of Health Sciences, The University of Texas at El Paso, El Paso, TX 79968, USA; pfhassan@miners.utep.edu (P.F.H.); mgomez26@utep.edu (M.G.); 2 Department of Physical Therapy and Movement Science, The University of Texas at El Paso, El Paso, TX 79968, USA; 3 Wu-Tsai Human Performance Alliance, Division of Cardiovascular Medicine, Stanford School of Medicine, Stanford University, Stanford, CA 94305, USA; smontal@stanford.edu

**Keywords:** arterial stiffness, stroke prevention, vascular function, ultrasound, tonometry

## Abstract

**Background:** The purpose of this investigation was to determine the elastic characteristics of the common carotid artery (CCA) during endurance exercise at 3 different intensities. **Methods**: Twenty young healthy participants (10 males and 10 females) participated in this quasi-experimental cross-sectional study. Participants were tested in two sessions: (1) we took resting measurements of the elastic characteristics of the CCA and performed a cardiopulmonary exercise test (CPET) on a cycle ergometer to determine submaximal exercise intensities, and we conducted (2) measurements of the elastic characteristics of the CCA while exercising in a cycle ergometer at 3 intensities based on blood lactate levels of low (<2 mmol/L), moderate (2–4 mmol/L), and high (>4 mmol/L). Beta stiffness was calculated using CCA diameters during systole and diastole, measured with high-definition ultrasound imaging, and CCA systolic and diastolic pressures were measured via applanation tonometry. **Results**: Overall, there were no differences between males and females in terms of any of the studied variables (*p* > 0.05). In addition, no significant changes were found in the CCA beta stiffness and vessel diameter (*p* > 0.05) between exercise intensities. There was a significant exercise intensity effect on CCA systolic pressure (*p* < 0.05), but not on CCA diastolic pressure (*p* > 0.05). **Conclusions**: The biomechanical characteristics of the CCA, determined via compliance and beta-stiffness, do not change during cyclical aerobic exercise, regardless of exercise intensity.

## 1. Introduction

The leading cause of death worldwide is cardiovascular disease (CVD), which includes coronary artery disease and stroke. The total direct and indirect costs of CVD in the USA are estimated to be around $43.6 billion [1]. Overall, 1 in every 19 deaths is caused by stroke in the USA, with more than 610,000 new cases of stroke per year. Of these, carotid artery (CA) atherosclerosis is responsible for 9 in every 10 cases of stroke [2]. Atherosclerosis happens when fat and cholesterol build up in the arteries or blood vessels, causing the arteries to narrow. Over time, the atherosclerotic plaque can break and bleed, causing an ischemic stroke. The rupture of CA plaque is also associated with biomechanical and hemodynamic changes in the CA. Therefore, understanding the elastic properties of the common CA (CCA), especially during endurance exercise, could help to prevent plaque rupture and stroke. 

Atherosclerotic plaque development is highly associated with endothelial shear stress [3,4,5]. Most of the plaque accumulation happens at the bifurcation of conduit arteries, such as the coronary and carotid arteries, associated with a decrease in the endothelial shear stress produced by turbulent flow at resting or baseline conditions [4,5,6,7]. However, this turbulent flow has also been shown to be vascular-protective, especially when endothelial shear stress is enhanced, like what happens during exercise [3,4,7,8]. In ischemic stroke, atherosclerotic plaque from the CA, most probably from the internal CA (ICA), ruptures and produces a clot that will travel to smaller arteries at the circle of Willis, producing an ischemic attack in the brain [9,10]. As both ICA and CCA are conduit arteries and have similar biomechanical characteristics [11,12], studying CCA during endurance exercise would enhance our understanding of atherosclerotic plaque ruptures.

Habitual exercise is an important factor in the condition of healthy arteries. For example, Tanaka et al. found that habitual endurance exercise decreased age-related arterial stiffness by using both cross-sectional and interventional approaches [13]. However, the same research group found that acute bouts of resistance exercise increased arterial stiffness after exercise [14]. In addition, Beck et al. [15] found that both resistance and endurance exercise training improved the arterial function of resistance arteries in young prehypertensive participants, with significant larger improvements seen with endurance training. It appears that resistance and endurance exercises might produce different effects in the arterial tree, and it is not clear what the changes are in CCA stiffness during an acute bout of endurance exercise. Studying the biomechanical characteristics of the CCA during exercise could further give a better insight into the interaction between exercise and the elastic component of the arterial tree. Furthermore, as Ogoh [16] and Liu et al. [17] recently described, it is important to differentiate the acute, accumulative, and chronic effects of exercise in the vasculature to better design exercise interventions for arterial stiffness and stroke prevention.

Therefore, the purpose of the current study was to determine the elastic characteristics of the CCA in vivo during acute endurance exercise at 3 different intensities. We hypothesize that arterial stiffness, measured via arterial compliance and beta stiffness index, will increase during exercise at higher intensities. This increase in stiffness at higher exercise intensities will be driven by a peripheral vasoconstriction.

## 2. Materials and Methods

### 2.1. Participant Characteristics

The present study was approved by the Institutional Review Board of The University of Texas at El Paso and conformed to the Declaration of Helsinki. All participants provided written informed consent before their participation in the study. Given that this is the first study attempting to analyze arterial compliance in the CCA during endurance exercise, an as a priori hypothesis was not generated, data were treated as exploratory, and a quasi-experimental cross-sectional study design was employed. Twenty apparently healthy young adults (*n* = 20; males = 10, females = 10) participated in this study. The inclusion criteria included no cardiovascular, metabolic, or neurological disease; blood pressure <120/80 mmHg; age 18–35 years old; and self-reported physical activity <150 min per week. Participants were required to abstain from food, caffeine, alcohol, non-steroidal anti-inflammatory drugs, and antioxidant supplementation for at least 8 h prior to testing, and 24 h abstinence from exercise. Premenopausal female participants were tested within 4 days before and after menses to standardize any hormonal influence on vascular function [18]. 

### 2.2. Experimental Design

Participants attended 2 testing sessions in the Clinical Applied Physiology (CAPh) Lab between 8 and 10 am: a (1) baseline testing session, and an (2) experimental testing session. During the baseline session, and after a 15 min resting period, participants laid down on an examination table to assess CCA diameters via high-definition ultrasound (MyLab30 Gold Cardiovascular, Esaote, Firenze, Italy). Additionally, we assessed CCA systolic and diastolic pressures by performing applanation tonometry (SphygmoCor CvMS, AtCor Medical, Sydney, Australia) on the left CCA. Ultrasound images were processed with an edge detection software (Vascular Imager 6 and Vascular Research Tools 6, Medical Imaging Applications LLC, Coralville, IA, USA) to determine systolic and diastolic diameters. CCA systolic and diastolic pressures were obtained in triplicate to ensure reliable data, with a variation of less than 5% in terms of coefficient of variation (CV) detected. The averages of these measures were recorded. Thereafter, participants performed a standardized cardiopulmonary exercise test (CPET) on a cycle ergometer with a graded exercise test protocol, including maximal oxygen consumption (VO_2_max, TrueOne 2400, Parvomedics Inc., Sandy, UT, USA), as previously described [3,7]. Heart rate (bpm, H7, Polar Electro Oy, Kempele, Finland), rate of perceived exertion (RPE, scale 7–20), and blood lactate levels (Lactate Plus, Nova Inc., Boston, MA, USA) were obtained at the last 10 s of each 2 min stages of the graded exercise protocol to determine the workloads for the exercise test during the experimental testing session. 

During the experimental testing session, participants completed three 5 min exercise bouts at 3 submaximal intensities, which were determined by the blood lactate levels during the CPET test during session 1: low-intensity < 2.0 mmol/L; moderate-intensity = 2.0–4.0 mmol/L; high-intensity > 4.0 mmol/L [3,7,19]. A nonlinear polynomial regression approach utilizing lactate (mmol/L) and exercise load (watts) from the baseline session was used to ensure that participants exercised at the intensities described above. During each exercise bout, a research assistant (MG) obtained two independent measurements of CCA diameters, via ultrasound imaging, and CCA systolic and diastolic pressures, as described earlier. The 2 measurements were obtained mid-way and during the last 30 s before the end of each bout. The average between both measurements was used for statistical analysis. 

### 2.3. Common Carotid Arterial Compliance and Beta Stiffness

Common carotid arterial compliance was calculated as follows: 

$Commoncarotidarterialcompliance=\frac{\left(Radial\;Strain\right)}{2*\left(CSP-CDP\right)}\times \pi CDP^{2}$, where CSP is the carotid systolic pressure, CDP is the carotid diastolic pressure, and Radial Strain is:

$Radial\;Strain=\frac{CSD-CDD}{CDD}$, where CSD is carotid systolic diameter and CDD is carotid diastolic diameter [14,20].

Beta stiffness was calculated as follows: 

$Beta\;stiffness=\frac{log(\frac{CSP}{CDP})}{Radial\;Strain}$ [13,14,21]

### 2.4. Statistical Analysis

Data were compiled into a Microsoft® Excel® (for Microsoft 365 MSO (Version 2404 Build 16.0.17531.20152) 64-bit) spreadsheet and then exported into RStudio Integrated Development Environment (IDE) for statistical analysis [22]. Descriptive statistics were obtained using the “psych” package. Data normality was assessed using a Shapiro–Wilk test. A linear mixed-effects model was utilized to determine carotid artery differences across intensities and sex using the “lmer” and “lme4” packages. Exercise intensity and the participant’s sex were included as fixed effects and, in addition, the participant was added as a random effect (slope and intercept). Thereafter, post hoc pairwise comparisons along with Cohen’s d and Hedge’s g correction were conducted where appropriate using the “rstatix” package. Lastly, as part of this exploratory study, all *p* values were reported. Data scripts are openly available at www.github.com/samuelmontalvo/carotid_compliance (accessed on 22 November 2023).

## 3. Results

All variables were found to be normally distributed. The characteristics of the sample are provided in Table 1. Males were taller, heavier, and hade higher resting systolic blood pressure than females (*p* < 0.05). No differences were observed in age, BMI, resting diastolic blood pressure, and VO_2_max between sexes. 

**Figure 1:**
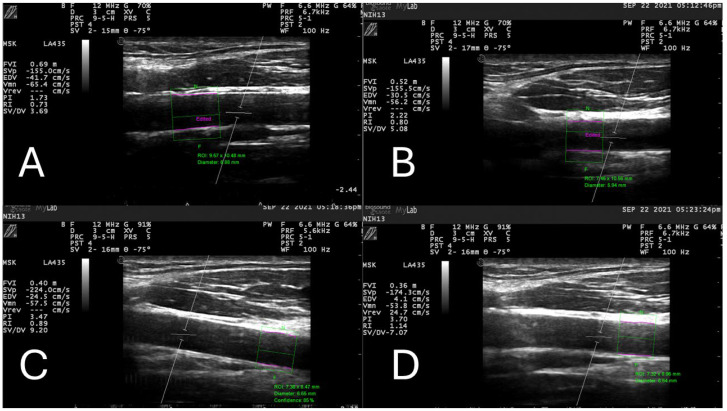
Representative ultrasound images and common carotid artery (CCA) diameter measurements of one subject during resting (**A**) and low-intensity (**B**), moderate-intensity (**C**), and high-intensity (**D**) exercise conditions. In all 4 images, the green dotted squares represent the region of interest (ROI), where the edge detection software (Vascular Research Tools, Medical Imaging Applications LLC, Coralville, IA, USA) identifies the edges of the CCA represented by the purple lines and measures the vessel’s diameter (values written in green letters).

Figure 1 shows representative ultrasound images and CCA diameter measurements, taken from one male participant during resting and low-, moderate-, and high-intensity exercise conditions. For this participant, the CCA diameters were 6.88 mm, 5.95 mm, 6.65 mm, and 6.64 mm for all four conditions, respectively. These values showed a similar trend to the general sample (Table 2).

The mixed models show no sex effect for markers of exercise intensity, blood pressure, carotid artery diameters, and arterial compliance and stiffness (Table 2). Almost all exercise intensity markers (workload, VO_2_, lactate levels, and RPE) showed full intensity dependency (*p* < 0.05 high vs. moderate, low and baseline; moderate vs. low and baseline; and low vs. baseline) in both males and females. HR showed partial intensity dependency (*p* < 0.05 high vs. moderate, low and baseline; moderate vs. baseline; and low vs. baseline in males and *p* < 0.05 high vs. low and baseline; moderate vs. low and baseline; and low vs. baseline in females). Regarding CCA pressures, in males, only systolic pressure showed full intensity dependency (*p* < 0.05 high vs. moderate, low and baseline; moderate vs. low and baseline; and low vs. baseline), and we detected partial intensity dependency in females (*p* < 0.05 high vs. moderate, low and baseline; moderate vs. baseline; and low vs. baseline), while diastolic pressures were only lower at baseline when compared to other exercise intensities in both males and females. Overall, both systolic and diastolic diameters did not increase or decrease during any of the exercise intensities, except for a small vasoconstriction between the baseline and low intensity in females (*p* < 0.05). Finally, there were no changes in terms of CCA compliance with exercise intensity in both males and females, and the CCA beta stiffness index showed a small increase between the baseline and moderate intensity in females (*p* < 0.05).

**Figure 2:**
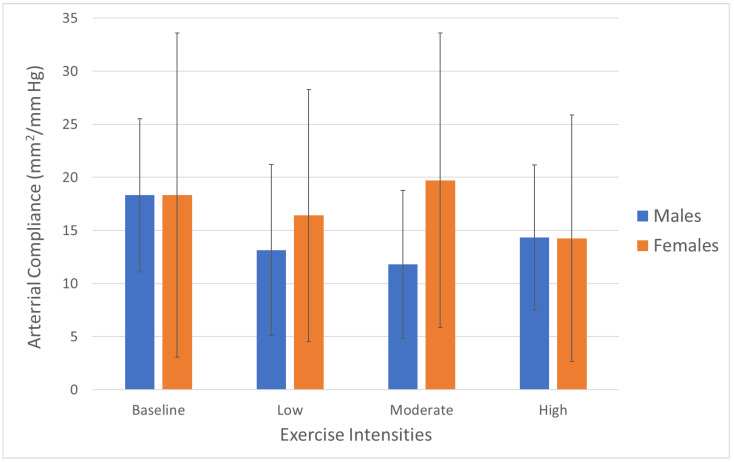
Common carotid arterial compliance by exercise intensity and sex. Data show means and standard deviations (*n* = 10/group).

**Figure 3:**
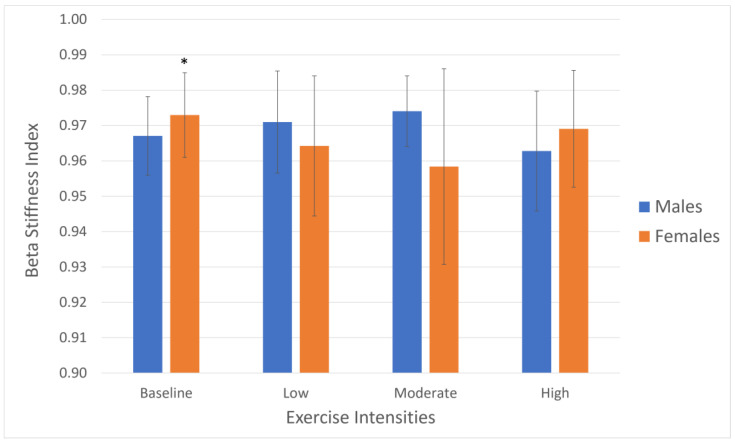
Common carotid artery beta stiffness index by exercise intensity and sex. Data show means and standard deviations (*n* = 10/group). * *p* < 0.05 baseline vs. moderate (females).

The individual mixed models showed no interaction between exercise intensity and sex in terms of arterial compliance (Figure 2) or beta stiffness (Figure 3). However, the CCA beta stiffness index showed that there was a small increase between the baseline and moderate intensity in females (*p* < 0.05, Figure 3).

As no sex effect was observed, we analyzed all variables as aggregate data (Table 3). All of the exercise intensity markers (e.g., workload, VO_2_, lactate, HR, and RPE) showed full intensity dependency (*p* < 0.05 high vs. moderate, low and baseline; moderate vs. low and baseline; and low vs. baseline). Regarding CCA pressure, systolic pressure showed full intensity dependency (*p* < 0.05 high vs. moderate, low and baseline; moderate vs. low and baseline; and low vs. baseline), while diastolic pressure was only lower at baseline when compared to all other exercise intensities. Interestingly, when aggregating the data from males and females, the systolic diameter showed some vasodilation during high-intensity exercise when compared with low-intensity exercise. Finally, there were no changes in diastolic diameter, CCA compliance, or beta stiffness index values with exercise intensity. 

## 4. Discussion

The purpose of this study was to explore the relationship between exercise intensity and carotid arterial compliance during cyclical aerobic exercise. The results of the current study showed that an acute bout of aerobic exercise does not produce any changes in the carotid artery’s diameters, compliance, or stiffness, being independent of exercise intensity and sex. Previous studies have shown changes in the elastic components of arteries after either chronic or acute exercise [13,14,23,24,25,26]; however, and based on our best knowledge, this is the first study showing the carotid artery’s biomechanical characteristics during an acute bout of exercise. 

The results of the current study are in agreement with previous reports [24,27]. Costa et al. [24] studied 19 post-menopausal females in a study with a cross-over design where they exercised acutely for 2 different days following a high-intensity interval exercise or moderate-intensity continuous exercise regimen. Large and small degrees of arterial compliance, measured via radial artery pulse wave analysis, were collected before and 30, 60, 90, and 120 min after the acute bout of exercise. The results showed no change in artery compliance, whether there was a large or small artery, between the baseline result and after exercise. In addition, Nickel et al. [27] studied 32 older males and females where they exercised for 30 min at 50% of their heart rate reserve. Large and small artery compliance, measured via diastolic pulse contour analysis, was collected before and every 15 min until 2 h after the acute bout of exercise. The results showed no change in artery elasticity in large arteries, with a non-significant decrease immediately after exercise. Small artery compliance showed an increase in elasticity from 30 to 75 min after exercise; however, elasticity decreased 2 h after exercise. Interestingly, DeVan et al. [14], using a similar approach to the current study by measuring the compliance and beta-stiffness of the CCA, showed a decrease in compliance and an increase in beta-stiffness immediately and 30 min after an acute bout of exercise. However, the acute exercise bout included 2 sets of 8–12 repetitions of 9 resistance exercise at 50% and 75% of the 1-RM. Recently, Liu et al. [17] performed an elegant study to determine the cumulative effects of exercise in terms of stiffness and hemodynamics in the CCA. The authors studied 30 healthy males in 4 different exercise conditions (no-exercise (CON); continuous exercise (CE, 30 min cycling); and accumulated exercise, including two or three bouts with 10 min rest intervals (AE15, 2 × 15 min cycling; AE10, 3 × 10 min cycling). Their main findings showed that there were no major differences immediately after (acute) any of the 3 exercise interventions; however, when exercise was extended in time (accumulated), there appeared to be an increase in cerebral blood supply. These results were similar to the ones in the current study, especially when compared with CE. Some of the differences between the studies could be attributed to the exercise intensity selection as Liu et al. used heart rate reserve, which is not very reliable [19,28]. Unfortunately, none of these studies can be fully compared to the current study. First, none of these studies measured arterial compliance during exercise. Secondly, not all studies used the same compliance assessment as the current study. Finally, not all studies used the same exercise modality. 

Interestingly, the study hypothesis was not confirmed. We expected to observe acute changes in arterial stiffness, driven by vasoconstriction, as observed in previous studies that assessed CCA after exercise [11,12]. The most plausible physiological explanation for these findings is that, during exercise, vascular and metabolic vasodilatory factors are able to enhance vasodilation over sympathetic vasocontraction or sympatholysis [29,30,31]. As soon as exercise is terminated, vascular and metabolic factors decrease their effects, but the sympathetic system stays active for longer, producing post-exercise vasoconstriction. The current study, when aggregating data from males and females, did show a trend of vasoconstriction during low-intensity exercise, most probably mediated by the sympathetic nervous system, which was compensated by a vasodilation during moderate and high intensities, most probably mediated by vascular factors such as endothelial shear stress. In addition, the current study did not find differences between sexes in any of the studied variables, which partially contradicted one of our previous studies [3]. Gurovich et al. [3] observed a systematic smaller brachial artery diameter in females compared to males during different exercise intensities and impaired sympatholysis in females at the highest intensity. These differences in the brachial artery’s diameter could be explained by differences in upper body muscle mass between sexes; however, brain mass and activity were the same in males and females, needing similar cerebral circulation.

Recently, Pierce et al. [32] and Saz-Lara et al. [33] published independent meta-analyses on the effects of an acute bout of exercise on arterial stiffness. In general, both studies showed that an acute bout of aerobic exercise does not produce significant changes in arterial stiffness after exercise. However, both studies had conflicting results when assessing acute resistance exercise and arterial stiffness. Pierce et al. [32] found that arterial stiffness increased after an acute session of resistance exercise. However, Saz-Lara [33] did not find any changes in arterial stiffness after an acute session of resistance exercise. These differences could be attributed to the different outcome measures the authors chose for their meta-analyses. Pierce et al. [32] used carotid–femoral pulse wave velocity (PWV), while Saz-Lara et al. [33] used a combination of central and peripheral PWV. Either way, the authors did not make any reference to arterial compliance, which is a more biomechanical characteristic than PWV. 

Exercise training is a well-documented intervention used to reduce arterial stiffness and improve cardiovascular risk factors [13,15,25]. For example, Beck et al. [15] showed that both resistance and aerobic exercise training can increase vascular conductance in patients with pre-hypertension. In addition, Tanaka et al. [13] showed that CCA compliance and beta-stiffness increased and decreased, respectively, after 3 months of endurance exercise training. Interestingly, Cortez-Cooper et al. [20] found no changes in CCA compliance after 13 weeks of resistance exercise training. The differences between the findings from Beck et al. [15] and Cortez-Cooper et al. [20], besides their outcome measures, might be explained by the population they studied. Patients with pre-hypertension had lower vascular conductance at baseline than normotensive controls [15], while participants who exercised in an apparently manner normal could have a ceiling effect showing no changes with training [20]. These differences were also found during acute bouts of exercise. For example, Costa et al. [23] found that larger decreases in arterial compliance after a 3 min step test were observed in subjects with the highest blood pressures. In addition, Tomoto et al. [26] found a lower CCA compliance in patients with traumatic brain injury when compared to age-matched controls, which tend to improve after 3 months of endurance exercise training. The current study included young, apparently healthy individuals and the findings during exercise also supported the possible ceiling effect. However, understanding the changes in biomechanical characteristics in the CCA during exercise can yield translational information for the prevention of stroke during exercise. 

It is important to consider that the participants were young, apparently healthy, and mostly sedentary, according to their VO_2_max (Table 1). Even though both females and males responded to the different exercise intensities similarly as previously reported by our CAPh Lab [19], males performed at a lower level than previously reported. It appears that males in the current study were not only less active, but also more overweight than females. However, and associated with the primary purpose of the current study, the lack of differences between sexes allowed us to merge the sample and analysis data as an aggregate, increasing the study’s power. 

The current study was not free of limitations. First, our sample size was limited to healthy asymptomatic young individuals. It remains unknown how the carotid artery responds during exercise in older individuals or patients recovering from a stroke. Secondly, the study design was focused on the effects during exercise and data from after exercise were not collected. This could give only a partial view of the exercise responses to cyclical endurance exercise. However, and as described above, there already are studies showing vascular compliance after exercise [14,23,24,26,27,32,33]. Nevertheless, the findings of the current study should be taken as exploratory and appropriate to the sample utilized in this study. Thirdly, the exercise sets in the current study were limited to 15 min using 3 different exercise intensities and it would have been interesting to assess the effects of longer sets as observed, during traditional exercise training bouts. Future studies could address this interesting question. Finally, and because of the nature of the study, we are not able to provide test–retest reliability measurements for intra-participant reliability; however, only one member of the team (MG) collected the data required to decrease this variability. These shortcomings should be addressed and taken into consideration by future studies. 

## 5. Conclusions

The biomechanical characteristics of the CCA, determined via compliance and beta-stiffness, do not change during cyclical aerobic exercise, regardless of exercise intensity. Further studies including older adults and stroke survivors should be conducted to make the current findings more transferable.

## Figures and Tables

**Table 1:** Participant characteristics.

	Overall (*n* = 20)	Female (*n* = 10)	Male (*n* = 10)	*p*
**Age (years)** **Mean (SD)**	26.9 (3.4)	26.3 (2.4)	27.4 (4.2)	0.48
**Height (m)** **Mean (SD)**	1.68 (0.10)	1.62 (0.06)	1.73 (0.10)	*0.01*
**Weight (kg)** **Mean (SD)**	68.9 (14.3)	62.2 (10.0)	75.7 (15.1)	*0.03*
**BMI (kg/m^2^)** **Mean (SD)**	24.5 (4.0)	23.6 (3.7)	25.3 (4.3)	0.38
**Resting systolic BP (mmHg)** **Mean (SD)**	113 (9)	109 (9)	117 (8)	*0.03*
**Resting diastolic BP (mmHg)** **Mean (SD)**	73 (14)	71 (11)	76 (17)	0.41
**VO_2_max (mL/kg/min)** **Mean (SD)**	27.6 (7.8)	28.1 (8.0)	26.9 (8.0)	0.76

BMI: body mass index; BP: blood pressure; VO_2_max: maximal oxygen consumption.

**Table 2:** Exercise markers, blood pressure, carotid artery diameter, arterial compliance, and arterial stiffness at each exercise intensity by Sex.

	Female				Male			
	Baseline (*n* = 10)	Low (*n* = 10)	Moderate (*n* = 10)	High (*n* = 10)	Baseline (*n* = 10)	Low (*n* = 10)	Moderate (*n* = 10)	High (*n* = 10)
**Workload (watts)** Mean (SD)	0.0 (0.0)	53.5 (17.0)	98.0 (34.7)	124.0 (38.4) ***	0.0 (0.0)	75.5 (39.0)	119.5 (42.7)	146.3 (42.8) ***
**VO_2_ (ml/kg/min)** Mean (SD)	4.16 (4.12)	15.83 (5.76)	20.76 (6.07)	26.93 (8.65) ***	3.57 (2.38)	12.85 (5.83)	20.27 (6.15)	23.62 (7.41) ***
**Lactate (mmol/L)** Mean (SD)	1.00 (0.39)	1.95 (0.87)	3.14 (0.89)	5.15 (1.91) ***	0.90 (0.44)	1.65 (0.53)	3.37 (1.40)	5.37 (2.26) ***
**Heart rate (bpm)** Mean (SD)	77 (18)	119 (15)	143 (24)	156 (11) *	77 (16)	104 (25)	132 (26)	144 (33) **
**RPE** Mean (SD)	6.1 (0.3)	9.0 (2.1)	12.2 (1.4)	14.9 (3.7) ***	5.4 (1.9)	7.5 (1.2)	10.9 (1.8)	13.9 (1.9) ***
**Car. Sys. pressure (mm Hg)** Mean (SD)	114 (14)	141 (20)	145 (18)	153 (15) **	119 (11)	138 (16)	151 (23)	159 (22) ***
**Car. Dias. pressure (mm Hg)** Mean (SD)	77 (10) †	83 (11)	88 (11)	88 (11)	80 (6) ††	83 (3)	88 (9)	88 (7)
**Diameter dystolic (mm)** Mean (SD)	6.19 (1.15)	5.90 (0.91)	6.07 (1.02)	6.05 (1.06)	6.62 (0.92)	6.56 (0.62)	6.70 (0.80)	6.74 (0.62)
**Diameter diastolic (mm)** Mean (SD)	5.85 (1.05) ‡	5.48 (0.82)	5.56 (0.76)	5.69 (1.08)	6.21 (0.95)	6.20 (0.95)	6.36 (0.76)	6.25 (0.63)
**Compliance (mm^2^/mm Hg)** Mean (SD)	18.34 (15.27)	16.41 (11.88)	19.72 (13.87)	14.26 (11.59)	18.31 (7.22)	13.16 (8.07)	11.79 (6.96)	14.34 (6.84)
**Beta stiffness (U)** Mean (SD)	0.972 (0.012) ‡‡	0.964 (0.020)	0.958 (0.028)	0.969 (0.017)	0.967 (0.011)	0.971 (0.014)	0.974 (0.010)	0.963 (0.017)

VO_2_: Oxygen consumption; RPE: Rates of perceived Exertion; Car. Sys.: Carotid Systolic; Car. Dias.: Carotid Diastolic; *** *p* < 0.05 high vs. moderate, low and baseline; moderate vs. low and baseline; and low vs. baseline; ** *p* < 0.05 high vs. moderate, low and baseline; moderate vs. baseline; and low vs. baseline; * *p* < 0.05 high vs. low and baseline; moderate vs. low and baseline; and low vs. baseline; † *p* < 0.05 baseline vs. low, moderate, and high (females); †† *p* < 0.05 baseline vs. low and moderate (males); ‡ *p* < 0.05 baseline vs. low (females); ‡‡ *p* < 0.05 baseline vs. moderate (females).

**Table 3:** Exercise markers, blood pressure, carotid artery diameter, arterial compliance and arterial stiffness at each exercise intensity including all participants.

	Baseline (*n* = 20)	Low (*n* = 20)	Moderate (*n* = 20)	High (*n* = 20)
**Workload** (watts)	0.0	64.5	108.8	135.1
Mean (SD)	(0.0)	(31.4)	(39.4)	(41.2) ***
**VO_2_ (mL/kg/min)**	3.83	14.17	20.49	25.09
Mean (SD)	(3.18)	(5.83)	(5.94)	(7.92) ***
**Lactate (mmol/L)**	0.95	1.80	3.26	5.26
Mean (SD)	(0.41)	(0.72)	(1.15)	(2.04) ***
**Heart rate (bpm)**	77	109	136	149
Mean (SD)	(16)	(22)	(25)	(27) ***
**RPE**	5.8	8.3	11.6	14.4
Mean (SD)	(1.4)	(1.8)	(1.7)	(2.9) ***
**Carotid systolic pressure (mm Hg)**	117	139	148	156
Mean (SD)	(13)	(18)	(20)	(18) ***
**Carotid diastolic pressure (mm Hg)**	78	83	88	88
Mean (SD)	(8) *	(8)	(10)	(9)
**Diameter systolic (mm)**	6.41	6.23	6.39	6.39
Mean (SD)	(1.04)	(0.83)	(0.95)	(0.92) **
**Diameter diastolic (mm)**	6.03	5.84	5.96	5.97
Mean (SD)	(0.99)	(0.82)	(0.84)	(0.91)
**Compliance (mm^2^/mm Hg)**	18.33	14.78	15.76	14.30
Mean (SD)	(11.62)	(10.03)	(11.43)	(9.26)
**Beta stiffness (U)**	0.970	0.968	0.966	0.966
Mean (SD)	(0.012)	(0.017)	(0.022)	(0.017)

VO_2_: Oxygen consumption; RPE: Rates of perceived Exertion; *** *p* < 0.05 high vs. moderate, low and baseline; moderate vs. low and baseline; and low vs. baseline; ** *p* < 0.05 high vs. low; * *p* < 0.05 baseline vs. low, moderate, and high.

## Data Availability

The raw data supporting the conclusions of this article will be made available by the authors on request.
